# Development and Validation of Robust Ferroptosis-Related Genes in Myocardial Ischemia-Reperfusion Injury

**DOI:** 10.3390/jcdd10080344

**Published:** 2023-08-12

**Authors:** Xiuxian Wei, Yi Li, Pengcheng Luo, Yue Dai, Tao Jiang, Mulin Xu, Yi Hao, Cuntai Zhang, Yu Liu

**Affiliations:** 1 Department of Geriatrics, Tongji Hospital of Tongji Medical College, Huazhong University of Science and Technology, Wuhan 430030, Chinahaoyi@hust.edu.cn (Y.H.); ctzhang0425@163.com (C.Z.); 2 Key Laboratory of Vascular Aging, Ministry of Education, Tongji Hospital of Tongji Medical College, Huazhong University of Science and Technology, Wuhan 430030, China; 3 Department of General Medicine, Tongji Hospital, Tongji Medical College, Huazhong University of Science and Technology, Wuhan 430030, China; 4 Department of Pathogen Biology, School of Basic Medicine, Tongji Medical College, Huazhong University of Science and Technology, Wuhan 430030, China

**Keywords:** myocardial ischemia-reperfusion, ferroptosis, bioinformatics analysis, immune infiltration, Hmox1

## Abstract

(1) Background: Despite the evidence that ferroptosis is involved in myocardial ischemia-reperfusion (MIR), the critical regulator of ferroptosis in MIR remains unclear. (2) Methods: We included three GEO datasets and a set of ferroptosis-related genes with 259 genes. Following the identification of the differentially expressed ferroptosis-related genes (DEFRGs) and hub genes, we performed the functional annotation, protein–protein interaction network, and immune infiltration analysis. The GSE168610 dataset, a cell model, and an animal model were then used to verify key genes. (3) Results: We identified 17 DEFRGs and 9 hub genes in the MIR samples compared to the control. Heme oxygenase 1 (Hmox1), activating transcription factor 3 (Atf3), epidermal growth factor receptor (Egfr), and X-box binding protein 1 (Xbp1) were significantly upregulated in response to ischemic and hypoxic stimuli. In contrast, glutathione peroxidase 4 (Gpx4) and vascular endothelial growth factor A (Vegfa) were consistently decreased in either the oxygen and glucose deprivation/reoxygenation cell or the MIR mouse model. (4) Conclusions: This study emphasized the relevance of ferroptosis in MIR. It has been successfully demonstrated that nine ferroptosis-related genes (*Hmox1*, *Atf3*, *Egfr*, *Gpx4*, *Cd44*, *Vegfa*, *asparagine synthetase* (*Asns*), *Xbp1*, and *bromodomain containing 4* (*Brd4*)) are involved in the process. Additional studies are needed to explore potential therapeutic targets for MIR.

## 1. Introduction

Ischemic heart disease is the leading cause of death worldwide, as revealed by the World Health Organization’s Global Health Estimates in 2019 [1]. Reperfusion therapies can effectively rescue ischemic tissue in the myocardium. However, reperfusion itself can trigger myocardial cell death and damage the function of cardiomyocytes, which is referred to as myocardial ischemia-reperfusion (MIR) or injury (MIRI) [2]. Mitochondrial dysfunction, activation of the cell death program, sterile inflammation, autoimmune response, calcium overload, and endothelial barrier dysfunction are all implicated in the MIRI pathological process. Unfortunately, current therapies, such as calcium channel antagonists, free radical scavengers, and antioxidants, show limited benefit in inhibiting MIRI. The underlying mechanism of MIRI is complex, and greater discovery detail remains critical for developing targeted therapies to reduce infarct size. Recently, ferroptosis has been suggested to be involved in MIRI, including iron overload and accumulation of reactive oxygen species (ROS) [3]. The specific role of ferroptosis in regulating MIR onset and development, however, remains largely unclear.

Ferroptosis is an iron-dependent form of regulated cell death characterized by lipid peroxidation, glutathione depletion, and inactivation of glutathione peroxidase 4 (GPX4) [4]. Impaired ferroptosis is implicated in regulating cardiovascular disease, and associated with various metabolic processes and inflammatory responses [5]. Ferroptosis has also been proven to be involved in the development of ischemia-reperfusion injury (I/R) [6]. During the reperfusion state, mitochondrial damage, electrolyte imbalance, and increased mitochondrial respiration trigger a burst of ROS, lipid peroxidation, and ferroptosis presented by a reduction in mitochondrial volume, reduced or even lost mitochondrial cristae, and condensed mitochondrial membrane densities. These morphological characteristics are distinguished from other forms of cell death, further highlighting the relevance of ferroptosis [7]. Moreover, the therapeutic strategy of targeting ferroptosis has been shown to attenuate pathological impairment in a variety of I/R models [8]. Alternative anti-ferroptosis agents are available; however, their precise mechanisms and potential downstream targets remain a mystery [3].

Therefore, we used three microarray gene expression datasets in the present study, including mouse and rat MIR cardiac tissues, to screen for the differentially expressed genes (DEGs) from public databases. These identified DEGs were then intersected with the ferroptosis dataset to obtain DEGs related to ferroptosis. These genes have been investigated for potential functional mechanisms and immune infiltration. We identified nine key ferroptosis-related genes in MIR based on a PPI network constructed using the STRING database and Cytoscape. These hub genes were verified using a test of independence of RNA-seq gene expression datasets, a model of oxygen and glucose deprivation/reoxygenation (OGD/R) injury in H9c2 cells, and a model of myocardial I/R injury in C57BL/6J mice. This study provides novel insights and potential therapeutic targets for treating ferroptosis in MIR.

## 2. Materials and Methods

### 2.1. Data Collection

The gene expression sets used in this study were downloaded from the Genes Expression Omnibus database (GEO) from the NCBI database (https://www.ncbi.nlm.nih.gov/geo/, accessed on 26 September 2022) with ‘myocardial ischemia reperfusion’ (MIR) as a keyword. Finally, three microarray datasets and one RNA-seq dataset were screened from GEO. In the GSE4105 dataset, there are six sham heart samples and six MI-R samples (reperfusion for 48 h (*n* = 3) or 7 days (*n* = 3)), which were performed on the GPL341 [9]. The dataset GSE61592 contains three sham and three MI-R heart samples (reperfusion for 72 h) performed on the GPL6887. In the GSE83472 dataset, four sham and four MI-R cardiac samples (reperfusion for 24 h) were run on the microarray platform GPL6885 [10]. In the GSE168610 dataset, four sham and four MI-R cardiac samples (reperfusion for 72 h) were carried out on the GPL19057 [11]. We used GSE4105, GSE61592, and GSE83472 to identify DEGs and differentially expressed ferroptosis-related genes (DEFRGs). We used GSE168610 for the validation of DEFRGs in subsequent analyses. The microarray data were log^2^ transformed and quantile normalized. The GEO datasets used in this paper can be found in Supplementary Table S1.

### 2.2. Analysis of RNA-seq Data

The RNA-seq data were obtained from the GEO database (GSE168610). The BiomaRt package was used for gene annotation, and the DESeq2 package was used to normalize read counts and analyze differential expressions. All genes were analyzed with principal component analysis (PCA) to determine their reproducibility (Supplementary Figure S1).

### 2.3. Identification of DEGs and DEFRGs

The identification of DEGs was performed using the Limma package in R software (version 4.2). A normal *p*-value < 0.05 and |log^2^FC| ≥ 0.05 were defined as the thresholds for selecting DEGs. A total of 259 ferroptosis-related genes (FRGs) were obtained from FerrDb (http://www.zhounan.org/ferrdb/legacy/, accessed on 26 September 2022). Seventeen overlapping DEFRGs were selected using the Venn Diagram in the R package.

### 2.4. Functional Enrichment Analyses

Gene Ontology (GO) enrichment of DEFRGs was analyzed using DAVID (Database for Annotation, Visualization, and Integrated Discovery). The GO analysis covers three areas: the cellular component (CC), the molecular function (MF), and the biological process (BP). Results with a *p*-value < 0.05 were statistically significant enrichments. Kyoto Encyclopedia of Genes and Genomes (KEGG) and Reactome enrichment analyses were used to predict the related pathways of the DEFRGs.

### 2.5. PPI Networks and Identification of Key Modules and Hub Genes

The protein–protein interaction (PPI) network of the DEFRGs was constructed according to the information acquired using the STRING database with the condition that the interaction combined score was >0.4 points. Cytoscape (version 3.9.2) was used to visualize the molecular interaction networks of the overlapping DEFRGs. CytoHubba, a Cytoscape plugin, was used to identify the hub genes in the PPI network. Of the 12 algorithms in CytoHubba, we randomly selected 5 (MCC, BottleNeck, EPC, EcCentricity, and Radiality). Each algorithm assigned a value for each gene and ranked those genes by their values. The top 10 genes were significant. We then took the intersection scores from the 5 algorithms to identify the hub genes.

### 2.6. Immune Infiltration Analyses

The proportion of infiltrating immune cells and the infiltration score were calculated with the CIBERSORT package in the R software (version 4.2). The results with *p* < 0.05 were defined as statistically significant differences.

### 2.7. Validation of Hub Genes in GSE168610

The RNA-seq GSE168610 dataset served as the independent dataset for validation purposes. After data preprocessing as described previously, the expression data of 9 hub genes were extracted, and groups were compared using the Wilcoxon test. Results with *p* < 0.05 were deemed to be statistically significant.

### 2.8. Cellular OGD/R Model

H9c2 rat myocardial cells were obtained from the American Type Culture Collection (ATCC, Manassas, VA, USA). Cells were cultured in high-glucose Dulbecco’s modified Eagle’s medium (DMEM, Gibco, Gaithersburg, MD, USA) supplemented with 10% fetal bovine serum (FBS, Gibco, Gaithersburg, MD, USA) and maintained in a humidified atmosphere consisting of 5% CO_2_ at 37 °C. After rinsing three times, the cells were incubated in DMEM without glucose and placed in a hypoxic incubator (1% O_2_, 94% N_2_, and 5% CO_2_) at 37 °C for 6 h. Subsequently, the glucose was replenished to normal levels, and the cells were incubated under standard growth conditions for 18 h.

### 2.9. ROS Detection

Dihydroethidium (DHE, HY-D0079, MedChemExpress, Monmouth Junction, NJ, USA) was used to detect ROS production in cultured cells. H9c2 cells were pretreated by the OGD/R model as described above, followed by incubation in DHE (10 µM) at 37 °C for 30 min in the dark. Images were captured using fluorescence microscopy. The fluorescence intensity was analyzed using Image-Pro Plus software (version 6.0). ROS-oxidized DHE in cells showed red.

### 2.10. Perls’ Prussian Blue Staining

Prussian Blue Iron Stain Kit (With Eosin Solution) (G1424, Solarbio, Beijing, China) was used to detect iron deposits. Briefly, for cardiac staining, sections were hydrated and rinsed in distilled water twice. Next, Perls’ staining working solution was incubated for 30 min. Sections were then stained with eosin and placed in ethanol solutions for rapid dehydration, followed by transparency and a sealing step. Tissues containing blue granules were considered Prussian blue staining positive. Images were taken under a light microscope.

### 2.11. Animals

C57BL/6J male mice (8 weeks old, 25 ± 1 g) were purchased from GemPharmatech., Ltd. (Nanjing, China) and were free of specific pathogens. Animals were housed in a temperature-controlled barrier facility with a 12 h light/dark cycle. All animal experiments were approved by Tongji Hospital, Tongji Medical College, Huazhong University of Science and Technology (Approval no: TJH-202110026). Procedures were performed following the Provision and General Recommendation of Chinese Experimental Animals Administration Legislation and the Guide for the Care and Use of Laboratory Animals (NIH Publication No. 85–23, revised 1996). Mice were numbered using an online number generator (http://www.randomizer.org, accessed on 25 October 2022) and then randomly divided into sham (Sham) and MIR groups. The sample size was calculated based on published studies using animal models [12].

### 2.12. Mouse Myocardial I/R Injury Model

Mice were anesthetized with an intraperitoneal pentobarbital injection. After intubation for ventilation, the heart was exposed through a left lateral thoracotomy. Cardiac ischemia was performed via the ligation of the left anterior descending coronary artery for 30 min using an 8-0 silk suture with a slipknot. Reperfusion was carried out by releasing the knot. Mice in the sham group received the same procedures without suture ligation. Forty-eight hours after reperfusion, mice were sacrificed following echocardiography, and cardiac tissue was harvested, which was either immediately snap-frozen in liquid nitrogen or fixed in 4% paraformaldehyde.

### 2.13. Transthoracic Echocardiography Measurement

Twenty-four hours after reperfusion, echocardiography and left ventricular (LV) hemodynamic measurements were performed using a Vevo 2100 System (FUJIFILM Visual Sonics, Toronto, Canada). Anesthesia was induced using 4% isoflurane mixed with 100% oxygen, and the mice were maintained under anesthesia using 2% isoflurane mixed with 100% oxygen. Ejection fraction (EF, %) and fractional shortening (FS, %) of the LV were assessed. The cardiac parameters were averaged from at least three separate cardiac cycles. Echocardiographic operators were blinded to mouse grouping.

### 2.14. Detection of MI/R Injury (TTC/Evans Blue Staining)

Triphenyltetrazolium chloride (TTC)-Evan’s Blue double stain was used to determine the size of the myocardial infarction. After reperfusion for 8 h, the 2% Evan’s Blue dye solution (E2129, Sigma, St. Louis, MO, USA) was injected into the peritoneal cavity to determine the area at risk of infection. Sixteen hours after injection, the mouse was sacrificed, and the heart was rapidly excised and rinsed in 0.9% saline and snap frozen at −20 °C for a further 10 min. LV was then cut into five slices of 1 mm thickness and incubated in 2% TTC (T8877, Sigma, St. Louis, MO, USA) for 30 min at 37 °C to determine the area of the infarcted area (white) as well as the viable areas (red). The infarct size ratio was measured as a percent of the total surface area.

### 2.15. Histological Analysis

The hearts were excised and postfixed in 4% paraformaldehyde, embedded in paraffin, and sectioned at 5 μm. Following deparaffinization and dehydration, the sections were stained with hematoxylin and eosin (HE) and observed under a light microscope (ML31, Guangzhou, China). One pathologist was blinded to MIRI grade. Histological analysis of infarct size, hemorrhage, and leukocyte infiltration was scored as none, weak, moderate, strong, or very strong (score 0, 1, 2, 3, or 4) [13].

Immunohistochemistry (IHC) staining was carried out using the following antibodies: activating transcription factor 3 (Atf3, YT0387, 1:250, Immunoway, Suzhou, China) and heme oxygenase 1 (Hmox1, ab68477, 1:200, Abcam, Cambridge, UK). For the IHC score, three fields from each sample were selected at random, and the mean score of the 3 fields was the final score [13]. An immunohistochemical assessment was performed by two investigators blinded to treatment assignment.

Immunofluorescent (IF) staining was carried out using the Hmox1 antibody (ab68477, 1:200, Abcam, Cambridge, UK). Images were captured on a confocal laser microscope (Nikon C2+, Tokyo, Japan).

### 2.16. Quantitative Real-Time PCR (qRT-PCR)

After RNA extraction, RNA quality and quantity were measured using a NanoDropTM^2000^ (Thermo Fisher Scientific, Inc., Waltham, MA, USA). The HiScript RT Kit (R222, Vazyme, Nanjing, China) was used to reverse the total RNA into cDNA. qRT-PCR was performed on a Step-One-Plus Real-time PCR system (Applied Biosystems; Thermo Fisher Scientific., Waltham, MA, USA) using ChamQ SYBR qPCR Mix (Q711, Vazyme, Nanjing, China). The PCR primers are shown in Supplementary Table S2, and qRT-PCR was performed as described previously [14]. Each assay was performed more than 3 times. The expression level was calculated with the 2^−ΔΔCt^ method and normalized to the Actb expression level, defined as 1.0.

### 2.17. Western Blot

The detailed Western blotting assay procedure was previously described [14]. The primary antibodies and their dilution ratios used for Western blotting were as follows: Hmox1 (ab68477, 1:1000, Abcam, Cambridge, UK), Atf3 (ab207434, 1:1000, Abcam, Cambridge, UK) and β-Tubulin (ABL1030, 1:4000, Abbkine, Wuhan, China). After incubation with appropriate HRP-conjugated secondary antibodies (1:5000, Santa Cruz Biotechnology, Santa Cruz, CA, USA) for 1 h at room temperature, the membranes were developed with an enhanced chemiluminescence reagent (Abbkine, Wuhan, China). The relative intensity of protein signals was normalized to the corresponding β-Tubulin intensity and was quantified using ImageJ software.

### 2.18. Glutathione (GSH) and GSH to Oxidized Glutathione Ratio (GSH/GSSG) Measurement

The total glutathione/oxidized glutathione assay kit (A061-1, Nanjing JianCheng Institute of Bioengineering, Nanjing, China) was used to detect total glutathione (T-GSH) and GSSG content. LV tissues (45 mg) were obtained from each sample (*n* = 3), and protein concentration was measured. According to the manufacturer’s instructions, the reagents and samples were added sequentially, and the 96-well plate was shaken before being placed in the microplate reader. The absorbance value A1 at 405 nm was read for 30 s and allowed to stand at room temperature (25 °C) for 10 min, and then, the absorbance value A2 at 405 nm was read for 10 min and 30 s. The contents of T-GSH and GSSG were calculated according to the standard curve. Reduced GSH level was calculated by the formula: reduced GSH = T-GSH − 2 × GSSG. The GSH/GSSG ratio was determined as follows: GSH/GSSG = (reduced GSH)/GSSG.

### 2.19. Flow Cytometry and FACS Sorting of Heart

For flow cytometry, a single-cell suspension was prepared as previously described [15]. Briefly, the heart was perfused with cold PBS and removed from the mouse. The heart was minced and digested with 400 U/mL collagenase Type II (LS004176, Worthington, Lakewood, NJ, USA) and 48 U/mL DNase I (DN25, Sigma-Aldrich, St. Louis, MO, USA) on a shaker at 37 °C for 40 min. Then, the cell digestion was filtered with a 70-μm strainer and centrifuged at 200× *g* for 5 min at 4 °C. Red blood cells were lysed with Red Blood Cell Lysis Buffer (R1010, Solarbio, Beijing, China) and washed with PBS. Before staining with the antibodies, the Fc receptors were blocked with mouse Fc block (anti-mouse CD16/32 Antibody, #553141, 1:100, B.D. Biosciences, San Jose, CA, USA) for 15 min on ice. Then, cells were stained with the Fixable Viability Stain 700 (#564997, 1:800, B.D. Biosciences, San Jose, CA, USA) on ice in the dark for 30 min to distinguish the live and dead cells. The fluorophore-conjugated antibodies used for analysis were as follows: CD45-APC-Cy7 (#557659, 1:100, B.D. Biosciences, San Jose, CA, USA), CD19- PerCP-Cy5.5 (#551001, 1:100, B.D. Biosciences, San Jose, CA, USA), CD64-BV786 (#741024, 1:100, B.D. Biosciences, San Jose, CA, USA), CD86-PE-Cy7 (#560582, 1:100, B.D. Biosciences, San Jose, CA, USA), and CD206-BV421 (#141717, 1:80, Biolegend, San Diego, CA, USA). Gates were set with the help of blank control and single staining. After extensive washing, cells were analyzed with FACS Cytoflex (Beckman Coulter, Brea, CA, USA), and analysis was performed using FlowJo software (version 10.0).

### 2.20. Statistical Analysis

SPSS Statistics (version 21.0) and GraphPad software (version 6.0) were used for statistical analysis and drawing. Continuous variables were expressed as mean ± SEM. The Shapiro–Wilk test was used to assess the normality of the data distribution. An unpaired Student’s *t*-test for two groups was performed for normally distributed data. The Mann–Whitney/Wilcoxon test for two groups was performed for non-normal distributed data. The significance level was set at a *p*-value < 0.05. The detailed statistical information is listed in Supplementary Table S7.

## 3. Results

### 3.1. The Overall Study Protocol and Identification of DEGs and DEFRGs

**Figure 1:**
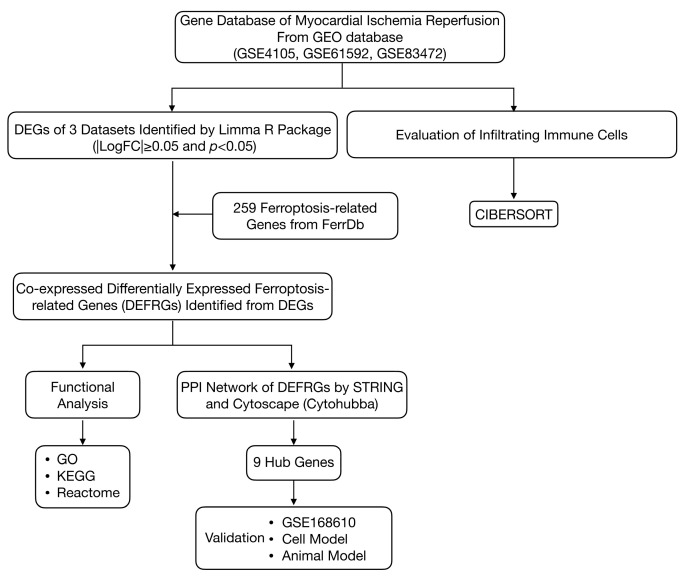
The overall protocol of this study. DEGs, differentially expressed genes.

**Figure 2:**
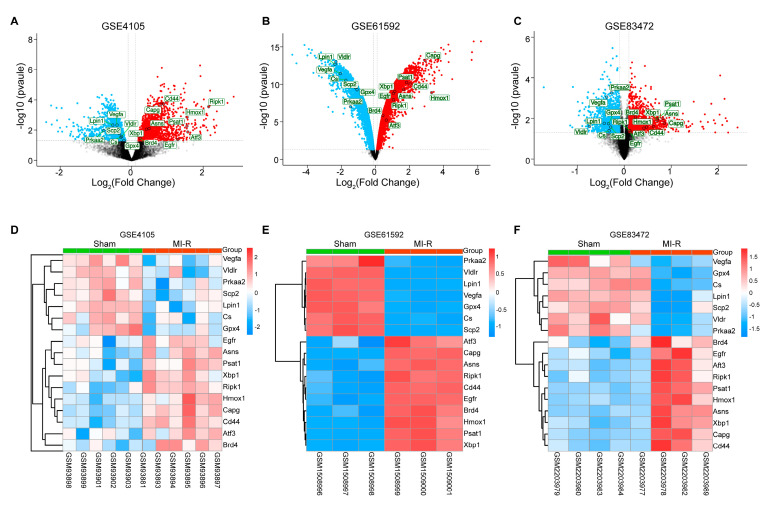
Identification of DEGs and DEFRGs. (**A**–**C**) Volcano plot of DEGs in GSE4105, GSE61592 and GSE83472, respectively (*p*-value < 0.05 and |log^2^FC| ≥ 0.05). (**D**–**F**) Clustered heatmap of DEFRGs in GSE4105, GSE61592, and GSE83472, respectively. DEGs, differentially expressed genes. DEFRGs, differentially expressed ferroptosis-related genes.

Figure 1 shows the overall flowchart for the study. DEFRGs were screened as markers, drivers, and suppressors of ferroptosis via the FerrDb online tool (Supplementary Table S3). After standardization of the microarray data, there were 2920 DEGs in GSE4105 (1477 upregulated and 1443 downregulated genes), 10,919 DEGs in GSE61592 (5585 upregulated and 5334 downregulated genes), and 3476 DEGs in GSE83472 (1745 upregulated and 1731 downregulated genes). The volcano plots are shown in Figure 2A–C. Then, the DEG sets intersected with 259 FRGs downloaded from the FerrDb database. Ultimately, 17 co-expressed DEFRGs were obtained, which contained 10 upregulated genes and 7 down-regulated genes (Supplementary Table S4). The clustered heatmaps of DEFRGs in the MI-R datasets are shown in Figure 2D–F.

### 3.2. Functional Enrichment Analysis of DEFRGs

**Figure 3:**
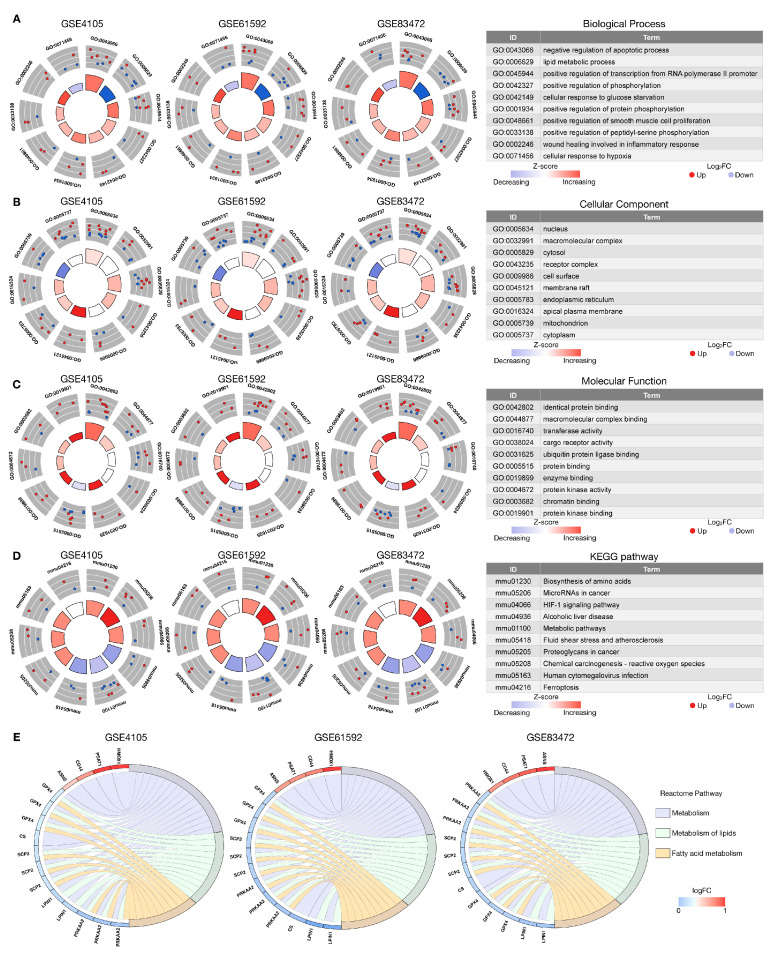
GO, KEGG, and Reactome enrichment analyses of DEFRGs. (**A**) The GO enrichment analysis of DEFRGs in the biological process (BP) category in GSE4105, GSE61592, and GSE83472, respectively, and the top 10 enriched GO-BP. (**B**) The GO enrichment analysis of DEFRGs in the cellular component (CC) category in GSE4105, GSE61592, and GSE83472, and the top 10 enriched GO-CC. (**C**) The GO enrichment analysis of DEFRGs in the molecular function (MF) category in GSE4105, GSE61592, and GSE83472, and the top 10 enriched GO-MF. (**D**) The KEGG enrichment analysis of DEFRGs in GSE4105, GSE61592, and GSE83472, and the top 10 enriched KEGG pathways; (**E**) The Reactome enrichment analysis of DEFRGs in GSE4105, GSE61592, and GSE83472.

GO, KEGG pathway, and Reactome pathway analyses were used to identify the biological functions of DEFRGs in the sham and MI-R samples (in GSE4105, GSE61592, and GSE83472) (Figure 3). GO enrichment analysis revealed that the BP was primarily enriched in negative regulation of the apoptotic process, lipid metabolic process, positive regulation of phosphorylation, cellular response to glucose starvation, wound-healing-involved inflammatory response, and cellular response to hypoxia (Figure 3A). The CC comprised the nucleus, macromolecular complex, cytosol, membrane raft, apical plasma membrane, and mitochondrion (Figure 3B). Furthermore, the MF contained identical protein binding, transferase activity, ubiquitin protein ligase binding, and enzyme binding (Figure 3C). The crucial KEGG pathways were ferroptosis, biosynthesis of amino acids, HIF-1 signaling pathway, reactive oxygen species, alcoholic liver disease, fluid shear stress, and atherosclerosis and metabolic pathways (Figure 3D). The Reactome enrichment analysis revealed that DEFRGs were linked to lipid metabolism and fatty acid metabolism (Figure 3E). This suggested that responses to ferroptosis-related metabolism and oxidative stress may play roles in regulating MI-R.

### 3.3. PPI Network and Identification of Hub Genes

**Figure 4:**
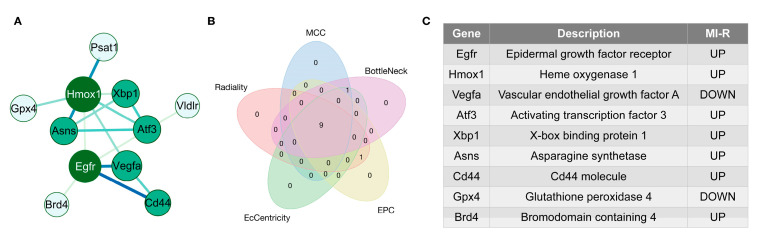
PPI network and identification of hub genes. (**A**) PPI network of DEFRGs with interaction combined scores > 0.4. (**B**) Venn diagram showing the overlap of the top 10 genes founded by the five algorithms in CytoHubba. (**C**) The nine key DEFRGs included seven upregulated and two downregulated genes in MI-R.

In this paper, five algorithms (MCC, BottleNeck, EPC, EcCentricity, Radiality) were adopted in CytoHubba to identify hub genes. To explore the interactions of these DEFRGs, we constructed a PPI network of DEFRGs with combined scores of >0.4 points using the STRING database, consisting of 11 nodes and 14 edges (Figure 4A). Six of the seventeen genes did not form a molecular network with other molecules. A list of the top 10 genes and the scores calculated by these five algorithms can be found in Supplementary Table S5. We then overlapped the top 10 genes found by all five algorithms to determine the core genes (Figure 4B,C). A total of seven upregulated genes (*epidermal growth factor receptor (Egfr)*, *Hmox1*, *Atf3*, *X-box binding protein 1* (*Xbp1*), *asparagine synthetase* (*Asns*), *Cd44*, and *bromodomain containing 4* (*Brd4*)) and two down-regulated genes (*vascular endothelial growth factor A* (*Vegfa*) and *Gpx4*) were chosen as the hub genes.

### 3.4. Immune Infiltration Analyses

**Figure 5:**
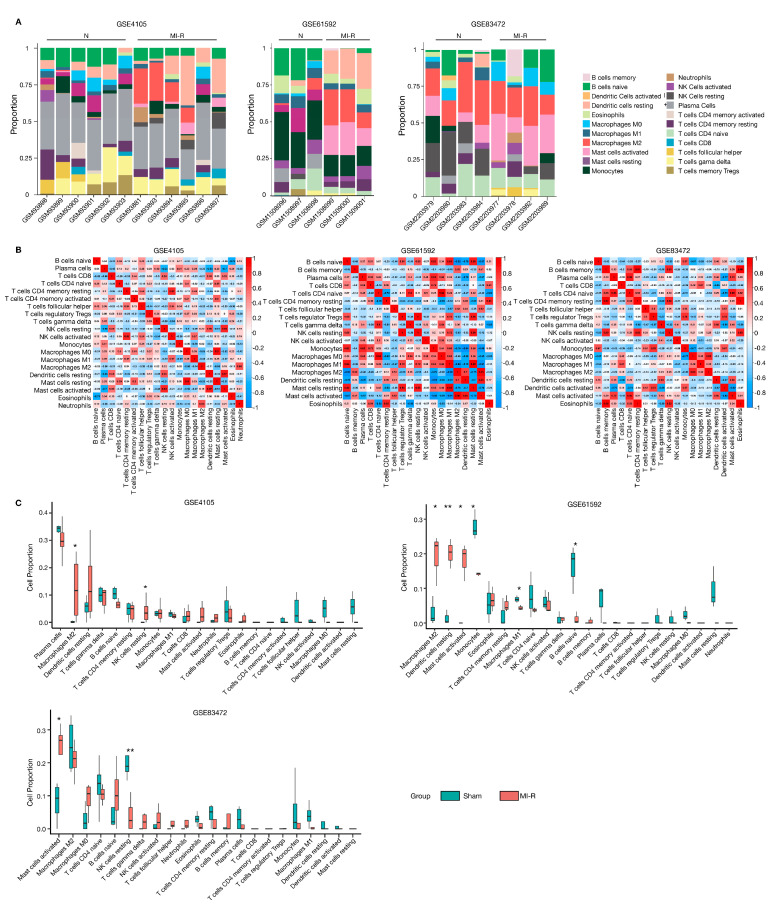
Immune infiltration analyses in GSE4105, GSE61592 and GSE83472. (**A**) The proportional histograms of the 22 immune cells calculated by the CIBERSORT algorithm in GSE4105, GSE61592, and GSE83472. (**B**) Clustered heatmap of immune cells in GSE4105, GSE61592, and GSE83472. (**C**) Differences in the proportion of immune cells in sham and MI-R heart samples in GSE4105, GSE61592, and GSE83472. Immune cells with a proportion of zero were hidden. * p < 0.05, ** p < 0.01.

The link between ferroptosis and inflammation has been widely reported [16]. We used the CIBERSORT package to analyze the infiltration of immune cells related to MI-R (extracted from GSE4105, GSE61592, and GSE83472). Figure 5A,B shows the proportions of 22 immune cell types and the correlations of the immune cells between the sham and MI-R groups. Specifically, we found a significant difference in the abundance of immune cells in GSE61592. Among all the different immune cells, regulatory T cells (Tregs) and resting NK cells had the highest positive correlation (Pearson’s correlation = 1). The second highest positive correlation was found between M2 macrophages and activated mast cells (Pearson’s correlation = 0.95). There was also a significant correlation between the naïve CD4 T cells and M0 macrophages (Pearson’s correlation = 0.94), as well as the M1 macrophages and resting mast cells (Pearson’s correlation = 0.93) (Figure 5B). The proportions of resting dendritic cells, activated mast cells, and M2 macrophages in the MI-R group were relatively high compared to those in sham tissue, whereas naïve B-cells, M1 macrophages, and monocytes were lower in GSE61592 (Figure 5C).

### 3.5. Validation of Hub Gene Expression and Immune Infiltration for MI-R Samples in GSE168610

**Figure 6:**
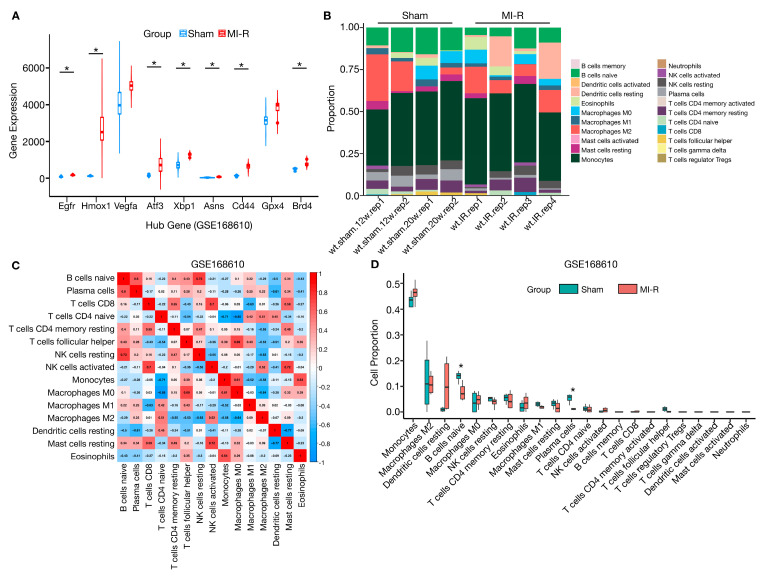
Validation of hub genes and immune infiltration analyses in GSE168610. (**A**) Expression of hub genes in sham and MI-R heart samples in GSE16810. (**B**) The CIBERSORT algorithm calculated the proportional histograms of the 22 immune cells in GSE168610. (**C**) Clustered heatmap of immune cells in GSE168610. (**D**) Differences in the proportion of immune cells in sham and MI-R heart samples in GSE168610. * p < 0.05.

To identify the reliability and accuracy of the bioinformatics analysis, GSE168610 was used to verify the expressional profiles of nine hub genes in MI-R cardiac samples via independent testing analysis. In summary, we found that the expression of Egfr, Hmox1, Atf3, Xbp1, Asns, Cd44, and Brd4 was significantly upregulated in the MI-R cardiac samples. Vegfa and Gpx4 did not reach statistical significance (Figure 6A, Supplementary Table S6). In addition, we analyzed immune infiltration in the GSE168610 dataset using the CIBERSORT software package (Figure 6B–D). Although there were some differences in the proportions of immune cell types, the differences did not reach significance. Additional statistical analysis showed that naïve B cells were significantly downregulated in the MI-R samples, which agreed with the GSE61592 results (Figure 6D).

### 3.6. Validation of Hub Gene Expression in the H9c2 OGD/R Model

**Figure 7:**
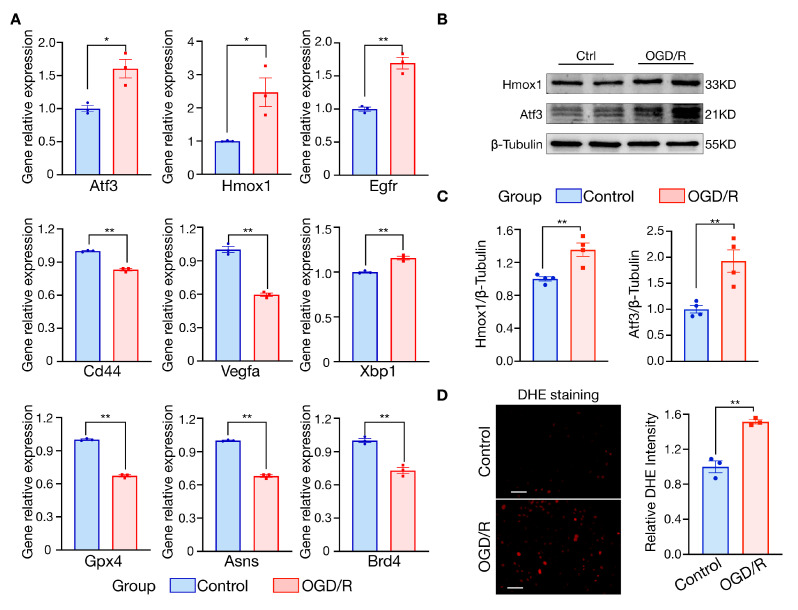
Validation of hub genes with OGD/R model in H9c2 cells. (**A**) Changes in mRNA expression of hub genes by qRT-PCR (*n* = 3). Data were shown as mean ± SEM. (**B**) Western blot of Hmox1, and Atf3 in control and OGD/R H9c2 cells. (**C**) Quantification of Western blot results (*n* = 4). Data were shown as mean ± SEM. (**D**) Representative images of DHE staining in H9c2 cells, quantification of the intensity of DHE fluorescence per field in control and OGD/R group (*n* = 3), scale bar = 50 μm. OGD/R, oxygen and glucose deprivation/reoxygenation. * p < 0.05, ** p < 0.01.

OGD/R is a standard cell model for myocardial ischemia-reperfusion injury, and ferroptosis has been identified as an essential mechanism of MIR injury in cardiomyocytes [17]. In this study, we constructed an OGD/R model of H9c2 cardiomyocytes and found significant changes in the mRNA expression of the nine hub genes (Figure 7). Based on the qRT-PCR results, these genes were consistent with the bioinformatic analysis described above, except for *Cd44*, *Asns*, and *Brd4* (Figure 7A). Consistently, the protein expression levels of Hmox1 (*p* = 0.0060) and Atf3 (*p* = 0.0064) were higher in the OGD/R group than in the control group (Figure 7B,C). Ferroptosis was implicated in OGD/R-induced cell death, as proved by DHE staining. There was a significant increase in the level of ROS in OGD/R H9c2 cells (Figure 7D).

### 3.7. Validation of Hub Gene Expression in MI-R Mice Heart Tissues

**Figure 8:**
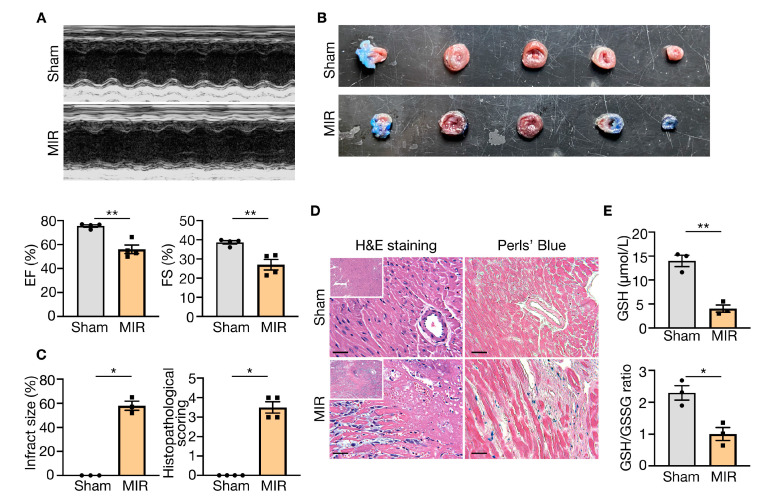
The occurrence of ferroptosis in myocardial I/R injury mice model. (**A**) Representative images of echocardiography and analysis of EF and FS in mice (*n* = 4). Data are shown as mean ± SEM. (**B**) TTC-Evans Blue-stained sections of hearts from each group. (**C**) (left) The infarct size of hearts was measured with TTC-Evans Blue staining (*n* = 3). The myocardial infarct size was expressed as the percentage of infarcts relative to the total area. (right) Histological analysis of HE staining (*n* = 4). Data are shown as mean ± SEM. (**D**) (left) Representative images of HE staining in two group, and (right) representative images of Perls’ Blue staining from each group, scale bar = 50 μm. (**E**) The content of GSH levels and GSH/GSSG ratio in heart tissues (*n* = 3). Data are shown as mean ± SEM. EF, ejection fraction. FS, fractional shortening. HE staining, hematoxylin-eosin staining. GSH, glutathione. GSSG, oxidized glutathione. * p < 0.05, ** p < 0.01.

**Figure 9:**
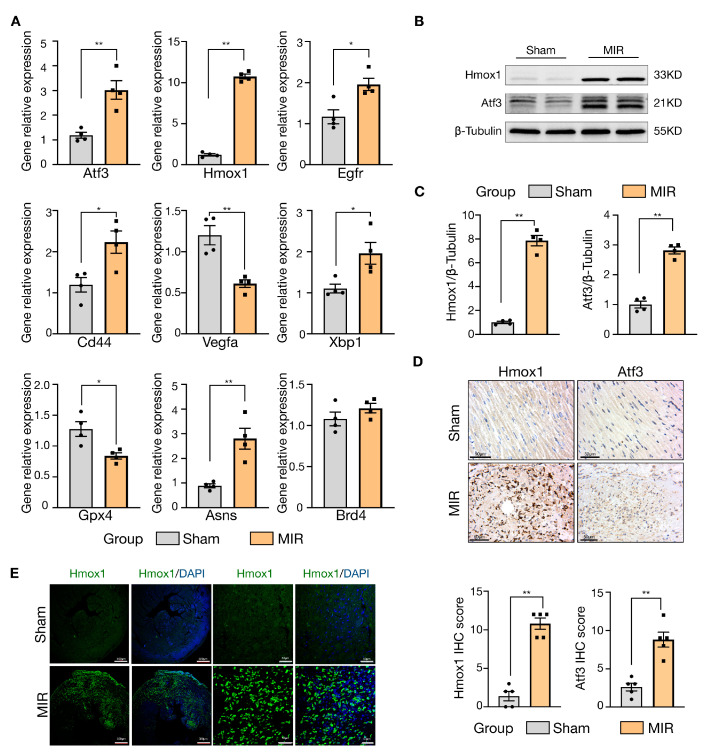
Validation of hub genes in MI-R mice myocardial tissues. (**A**) Changes in mRNA expression of hub genes by qRT-PCR (*n* = 4). (**B**) Western blot of Hmox1 and Atf3 in sham and MIR mice. (**C**) Quantification of Western blot results (*n* = 4). (**D**) IHC staining and IHC score analysis for Hmox1 and Atf3 in mice heart tissues (*n* = 5), scale bar = 50 μm. (**E**) Representative images of IF staining for Hmox1(green) and DAPI (blue) in mice heart tissues, scale bar = 500 μm (left) or 50 μm (right). Data are shown as mean ± SEM. * p < 0.05, ** p < 0.01.

We built an MI-R mouse model, which was shown to be successfully constructed using echocardiography and TTC/Evans blue staining. There was a significant decrease in left ventricular EF (*p* = 0.0019) and FS (*p* = 0.0067), as well as an increase in infarct size after myocardial I/R (Figure 8A,B). HE staining showed extensive myocardial injury, including disorder of myocardial structure, interstitial hyperemia, and edema (Figure 8C,D). Perls’ blue staining showed ferroptosis in the MIR lesion tissues (Figure 8D). We examined the GSH content and GSH/GSSG ratio between groups, since GSH is an index of the GSH-GPX4 axis and ferroptosis [18]. As a result, the GSH level (*p* = 0.0021) and GSH/GSSG ratio (*p* = 0.0135) in mouse LV tissue were lower in the MI-R group compared to the sham group (Figure 8E). The relative mRNA expression levels of *Atf3* (*p* = 0.0035), *Hmox1* (*p* < 0.0001), *Egfr* (*p* = 0.0126), *Cd44* (*p* = 0.0186), *Xbp1* (*p* = 0.0238), and *Asns* (*p* = 0.0044) were upregulated in the MI-R tissues compared to the sham group, while *Vegfa* (*p* = 0.0033) and *Gpx4* (*p* = 0.0156) were downregulated in the MI-R heart. This was accompanied by a significant increase in the protein expression levels of Hmox1 (*p* < 0.0001) and Atf3 (*p* < 0.0001), which was consistent with the results of the bioinformatic analysis (Figure 9A–C). Notably, the most significant change in the increase was in Hmox1 (*p* < 0.0001), which was 10 times higher compared to the control (Figure 9A–D), and Hmox1 was predominantly elevated in myocardial tissue (Figure 9E). Despite an increase in *Brd4*, it was insignificant (Figure 9A).

### 3.8. Validation of Immune Infiltration in MI-R Mice Heart Tissues

**Figure 10:**
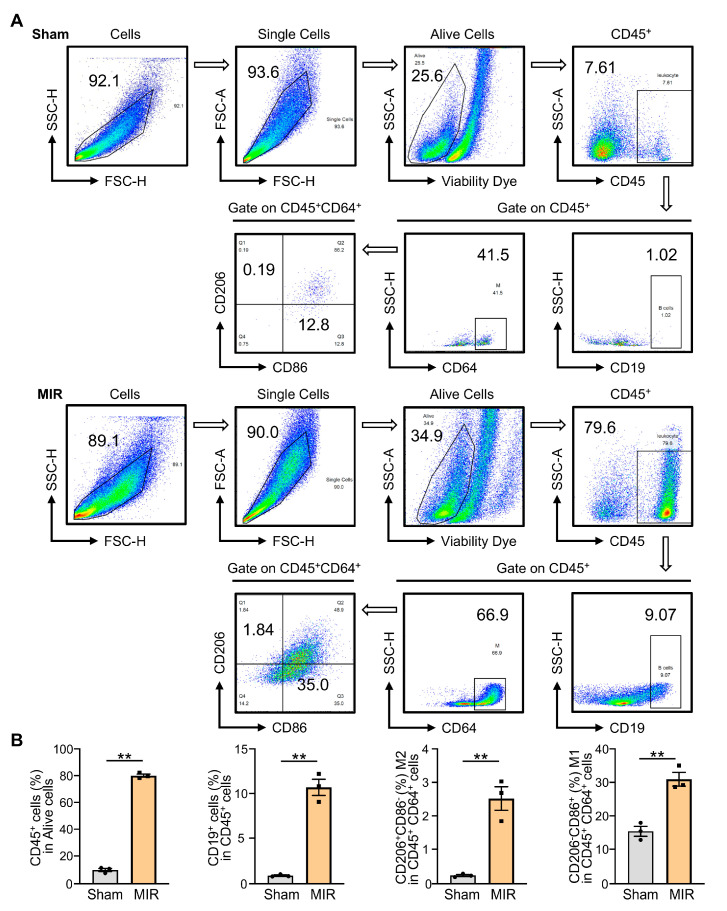
Validation of immune infiltration in the heart tissues of sham and MI-R mice. (**A**) FACS gating strategy to analyze different immune cells. (**B**) Flow cytometry analysis of CD45^+^ cells (leukocytes), CD45^+^CD19^+^ cells (B cells), CD206^+^CD86^−^ in CD45^+^CD64^+^ cells (M2 macrophages), and CD206^−^CD86^+^ in CD45^+^CD64^+^ cells (M1 macrophages) in cardiac tissues (*n* = 3). ** *p* < 0.01.

Next, we examined the immune infiltration in the hearts of sham and MI-R mice using flow cytometry (Figure 10A). Consistent with the results of the bioinformatic analysis, the infiltration of CD45^+^ leukocytes (*p* < 0.0001) and CD206^+^CD86^−^ M2 macrophages (*p* = 0.003) were significantly increased in MI-R hearts at 48 h after reperfusion compared to the sham group (Figure 10B). However, CD45^+^CD19^+^ B cells (*p* = 0.0004) and CD206^−^CD86^+^ M1 macrophages (*p* = 0.0035) were both increased in MI-R hearts, which is inconsistent with our bioinformatic prediction (Figure 10B).

## 4. Discussion

**Figure 11:**
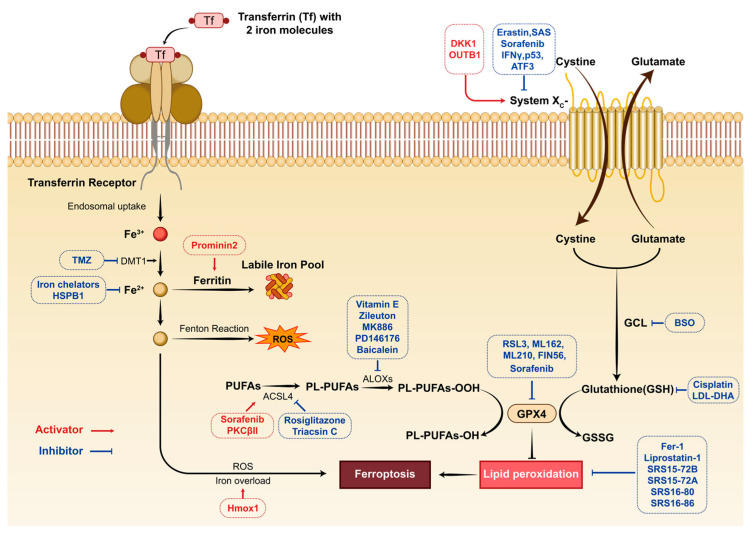
Key molecules in the ferroptosis pathway and therapeutic strategies of genetic/pharmacological activators and inhibitors targeting ferroptosis. The initiation and execution of ferroptosis are primarily influenced by iron metabolism, lipid peroxidation, and the dynamic balance of the GSH/GPX4 axis. Extracellular Fe enters the cell through the transferrin receptor and triggers the Fenton reactions to generate ROS. Lipid peroxidation occupies a unique position in ferroptosis. PUFA participates in lipid peroxidation through a variety of enzyme catalysis (such as ACSL4 and ALOXs), which are important steps in promoting ferroptosis. Due to its role in regulating ROS accumulation and oxidative stress, the GSH-GPX4 axis is a broad antioxidant system that protects cells from ferroptosis. The development of ferroptosis-inducing/inhibiting agents shows great potential in treating various diseases. TMZ, temozolomide; DMT1, divalent metal transporter 1; HSPB1, N-terminus of small heat shock protein 1; ROS, reactive oxygen species; Hmox1, heme oxygenase 1; PUFAs, polyunsaturated fatty acids; PL-PUFA, phospholipids-PUFA; ACSL4, acyl-CoA synthetase long chain family member 4; ALOXs, arachidonate lipoxygenases; GPX4, glutathione peroxidase 4; RSL3, RAS synthetic lethal 3; FIN56, ferroptosis inducing 56; DKK1, dickkopf-1; OTUB1, ovarian tumor domain containing ubiquitin aldehyde binding protein 1; SAS, sulfasalazine; IFNγ, Interferon γ; ATF3, activating transcription factor 3; GCL, glutamate–cysteine ligase; BSO, buthionine sulfoximine; LDL-DHA, low density lipoprotein-docosahexaenoic acid nanoparticles; GSSG, oxidized glutathione; Fer-1, ferrostatin-1.

Ferroptosis is a novel form of programmed cell death dependent on iron and distinct from apoptosis and necrosis [4]. The major morphologic manifestations of ferroptosis are mitochondrial abnormalities, and biochemical features primarily include iron and ROS accumulation, depletion of GSH, arachidonic acid release, and inhibition of the cystine/glutamate pathway (System Xc^−^) [2] (Figure 11). Regulatory inhibition or activation of this death process has become a critical research field since ferroptosis can potentially be developed into treatments for many diseases, such as tumors, renal injuries, and blood disorders [19] (Figure 11). The critical role of ferroptosis in cardiovascular disease has also been reported in prior studies, including studies of MIRI [5]. Inhibiting ferroptosis could be a promising therapeutic strategy for ischemic-reperfusion [20]. A remarkable paradigm has emerged for treating ischemia-reperfusion injury with sodium-glucose cotransporter-2 inhibitors (SGLT2i), such as empagliflozin [21]. Dapagliflozin, as another SGLT2i, protects the myocardium against ischemia-reperfusion injury by reducing ferroptosis and targeting MAPK signaling [22]. Another evidence comes from the successful cardioprotective role of Fingolimod, a sphingosine-1-phosphate (S1P) receptor agonist, in ameliorating ischemia-reperfusion injury [23], since S1P has been recently shown to alleviate radiation-associated ferroptosis by promoting the expression of GPX4 in ovarian tissue [24].

In a recent bioinformatic analysis, seven genes associated with ferroptosis in the pathogenesis of MI/R were identified from a GEO dataset with a rat MIRI model (GSE4105) and successfully verified under hypoxia/reoxygenation conditions [25]. However, there is a greater focus on integrating data assets because signal source bias and single species limit the power of data. In this study, we filtered 17 DEFRGs identified from overlapping DEGs in three datasets from GEO (including mice and rat samples) and FerrDb. These DEFRGs included 10 genes upregulated and 7 genes downregulated in MIR samples relative to healthy heart samples. Then, nine hub genes (*Egfr*, *Hmox1*, *Atf3*, *Xbp1*, *Asns*, *Cd44*, and *Brd4* upregulated; *Vegfa* and *Gpx4* downregulated) showed high scores in the five algorithms of CytoHubba in the PPI network. Immune infiltration analysis showed that key genes were associated with immune cells following MIRI. We then validated the hub genes in GSE168610, and the expression of these genes, except for Vegfa and Gpx4, was consistent with the results of the bioinformatics analysis. The significance of those genes was further validated in the H9c2 OGD/R model and the MI/R mouse model. Based on the results, the expression of all nine hub genes agreed with the bioinformatic analysis result.

Both innate and adaptive immune responses are required for the development and repair of MIRI [26]. Given the importance of ferroptosis in regulating immunity [27], it is reasonable to convince that an imbalance in iron homeostasis can trigger immune cell death, expanding the damaged size following MIR. After myocardial infarction, ferroptosis-induced neutrophil infiltration is involved in developing myocardial injury [28]. As another predominant cell type during MIR, macrophages exhibit functional heterogeneity, with proinflammatory macrophages (M1 macrophages) initially infiltrating, followed by a second wave of anti-inflammatory macrophages (M2 macrophages) in response to MIRI [29]. Our bioinformatic analysis showed that M2 macrophages, resting dendritic cells, and activated mast cells were more prevalent in MIR tissue than in the sham group. In contrast, M1 macrophages, monocytes, and naive B cells were found to be decreased in MIR cardiac tissue. We examined the immune infiltration by FACS and found leukocyte infiltration (including M1 and M2 macrophages) in the MIRI heart. This may be explained by the fact that circulating monocytes can differentiate into macrophages as early as one day after myocardial infarction [30]. M1 macrophages exerted a higher resistance to ferroptosis than M2 macrophages [31]. Thus, ferroptosis may contribute to the depletion of M2 macrophages, which can cause tissue damage and even cardiac rupture in the reparative phase. The sample-collection time may be another factor that affect the results. Cardiac samples were obtained after 30–90 min of ischemia, followed by 1 to 7 days of reperfusion. As a result, it is challenging to eliminate discrepancies between experiments. MIR is an inflammatory process that involves ferroptosis and immunity, yet its exact mechanisms remain unclear.

According to studies, all hub genes are involved in ferroptosis depending on disease and condition, with Egfr and Atf3 acting as ferroptosis drivers and Gpx4, Cd44, and Brd4 as inhibitors. The role of Hmox1 in ferroptosis is twofold. Hmox1 is a cytoprotective stress protein responsive to oxidative and inflammatory stimuli [32]. Transgenic mice overexpressing Hmox1 in the cardiomyocyte showed a limited infarction area and decreased inflammation after MIR compared to the wild-type group [33]. However, Hmox1 can produce ferrous iron by degrading heme, causing mitochondrial iron overload and increasing ferroptosis in cardiomyocytes [34]. Machado et al. demonstrated that following injury, deletion of the myocardial ferritin heavy chain led to a compensatory up-regulation of Hmox1, causing Slc7a11 overexpression and protecting the heart from ferroptosis mediated by ischemia-reperfusion [35]. It is consistent with our findings that Hmox1 increased in the OGD/R cell model and MI/R mouse model. Specifically, immunofluorescent histochemical staining showed that Hmox1 was primarily enriched in the region of infarction (Figure 9). Hmox1-induced cardioprotection may be enhanced with ferroptosis inhibitors co-administered concomitantly by reducing ferroptosis’ detrimental aspects [36]. Thus, therapeutic strategies involving alterations in Hmox1 activity for MIR should take into account the risk of ferroptosis.

Our data revealed that Atf3, Egfr, and Xbp1 were upregulated in cells and mice in response to ischemic and hypoxic stimuli. As a member of the ATF/cyclic adenosine monophosphate response element-binding family, Atf3 can promote ferroptosis by depleting intracellular GSH via inhibiting the system Xc^−^ [37]. On the other hand, ATF3 induced transcriptional activation of FANCD2 to suppress cardiac ferroptosis [38], raising the possibility that there may be a negative feedback autoregulatory mechanism in ATF3-related ferroptosis. It is unclear, however, whether this autoregulation of Atf3 is involved in the MIR stress response. By comparison, the role of Egfr in ferroptosis is relatively clear. Egfr is a classical receptor tyrosine kinase. Upon cystine depletion, EGFR can sensitize cells to ferroptosis [39]. EGFR inhibition might promote cardiomyocyte survival in hypoxia by combining genetic and chemical screens [40]. According to our findings, the downregulation of Egfr may abrogate the deleterious effects of ferroptosis and protect cardiocytes against MIR. Ferroptosis is related to endoplasmic reticulum (ER) stress, and activating the ER stress pathway worsens ferroptosis in certain diseases [41]. Given the vital role of Xbp1 in response to ER stress [42], it is reasonable that Xbp1 promoted ferroptosis in cerebral infarcts via activation of ER stress [43]. However, Xbp1 activation during cardiac I/R injury helps to reduce MIRI [44]. Our results may provide new clues for reassessing the role of Xbp1 in MIRI. Further investigation is required to determine whether Xbp1 protects MIRI by inhibiting ferroptosis or by an alternative mechanism.

According to our observations, Gpx4 and Vegfa were consistently decreased in either the OGD/R cell model or the MI/R mouse model. It is well known that Gpx4 regulates the ferroptotic process as an endogenous antioxidant against lipid peroxidation [45]. Hetero deletion of Gpx4 aggravates myocardial infarct size of MIRI, and the overexpression of cytosolic Gpx4 suppressed cell death induced by OGD/R [36], which is in agreement with our data. Considering the significance of VEGF-A in angiogenesis, it is convincing that restoring the expression of VEGF-A contributes to recovering the blood supply to decrease I/R-induced myocardial infarction size [46]. Recent bioinformatics analyses suggest that Vegfa is an essential ferroptosis-related gene in sepsis, lupus nephritis, and spinal cord injury [47]. The role of Vegfa in ferroptosis needs to be further investigated, especially when applied to the MIR model.

The Cd44, Asns, and Brd4 genes were upregulated in MIR cardiac samples, while these were downregulated in the OGD/R H9c2 model, suggesting that the regulation of ferroptosis in the MIR may be multifaceted. Brd4 is an essential epigenetic regulator of genes related to fatty acid metabolism that affects ferroptosis. Inhibiting Brd4 weakened mitochondrial function and reduced lipid ROS formation, rendering cells insensitive to Erastin-induced ferroptosis [48]. Brd4 was significantly upregulated in MIRI, and inhibition of Brd4 ameliorated renal I/R injury and MIRI by reducing oxidative stress and neutrophil accumulation [49]. Besides, glutamine-dependent asparagine synthesis is catalyzed by Asns, an amino acid-responsive gene [50]. Asns was proposed as a potential protector against ferroptosis and irradiation based on amino acids’ role in ferroptosis [51]. The contradictory findings of Asns in cardiac tissues and H9c2 OGD/R cells may be due to the lack of in vivo feedback regulation in the development of the MIR. As a marker for reperfusion injury, Cd44 signaling regulates fibroblast function and is necessary for the resolution of inflammation after infarction [52]. For now, the role of Cd44 in both ferroptosis and the MIR remains unclear. It is important to note that the hub genes we have identified are not specific to ferroptosis but have also been implicated in other biological processes, perhaps beyond ferroptosis.

In the research, we highlighted the potential role of ferroptosis-related key genes in MIR and verified the expression of hub genes in another dataset as well as in cells and mice under hypoxic and ischemic stimuli. However, we acknowledge that this study has several limitations. First, the correlation between hub genes and ferroptosis in MIR was validated in in vitro and in vivo models. However, the regulatory mechanisms and the hierarchical network of these genes involved in ferroptosis and MIRI are still unclear and need further investigation. Second, the expression changes of hub genes and the immunological changes across different reoxygenation or reperfusion times should also be comprehensively evaluated. Third, the data used in our bioinformatics analysis were obtained from public databases, lacking suitable human sample datasets; therefore, additional validation of these hub genes is required in clinical settings.

## 5. Conclusions

In conclusion, we filtered 17 DEFRGs and identified 9 ferroptosis-related genes (*Hmox1*, *Atf3*, *Egfr*, *Gpx4*, *Cd44*, *Vegfa*, *Asns*, *Xbp1*, and *Brd4*) in MIR, and that the immune response may play a crucial role in MIRI. Our results suggested that these genes are involved in developing MIR through ferroptosis. Further studies are required to clarify the exact mechanisms and explore the potential therapeutic targets.

## Data Availability

The datasets presented in this study can be found in online repositories. The names of the accession number (s) can be found in the article/Supplementary Material. All data generated or analyzed during this study are available from the corresponding author on reasonable request.

## Supplementary Materials

The following supporting information can be downloaded at: https://www.mdpi.com/article/10.3390/jcdd10080344/s1, Figure S1: PCA plot of GSE168610 RNA-seq data according to gene expression level; Table S1: GEO datasets used in this article; Table S2: Primers of ferroptosis DEGs used for qRT-PCR; Table S3: Classification of the DEFRGs; Table S4: DEFRGs including 10 upregulated and 7 downregulated genes in MI-R; Table S5: Top 10 hub genes as ranked in CytoHubba; Table S6: P values of hub gene expression in GSE168610; Table S7: Statistical information.
