# Supplementary material for: Development and Validation of Robust Ferroptosis-Related Genes in Myocardial Ischemia-Reperfusion Injury
Source: J Cardiovasc Dev Dis. 2023 Aug 12;10(8):344. doi: 10.3390/jcdd10080344 (PMC10455596; doi:10.3390/jcdd10080344)
Supplement: Supplementary file 1 [file jcdd-10-00344-s001.zip › supplementary files/Additional file 1 (SF1).docx]

## Supplementary Figure


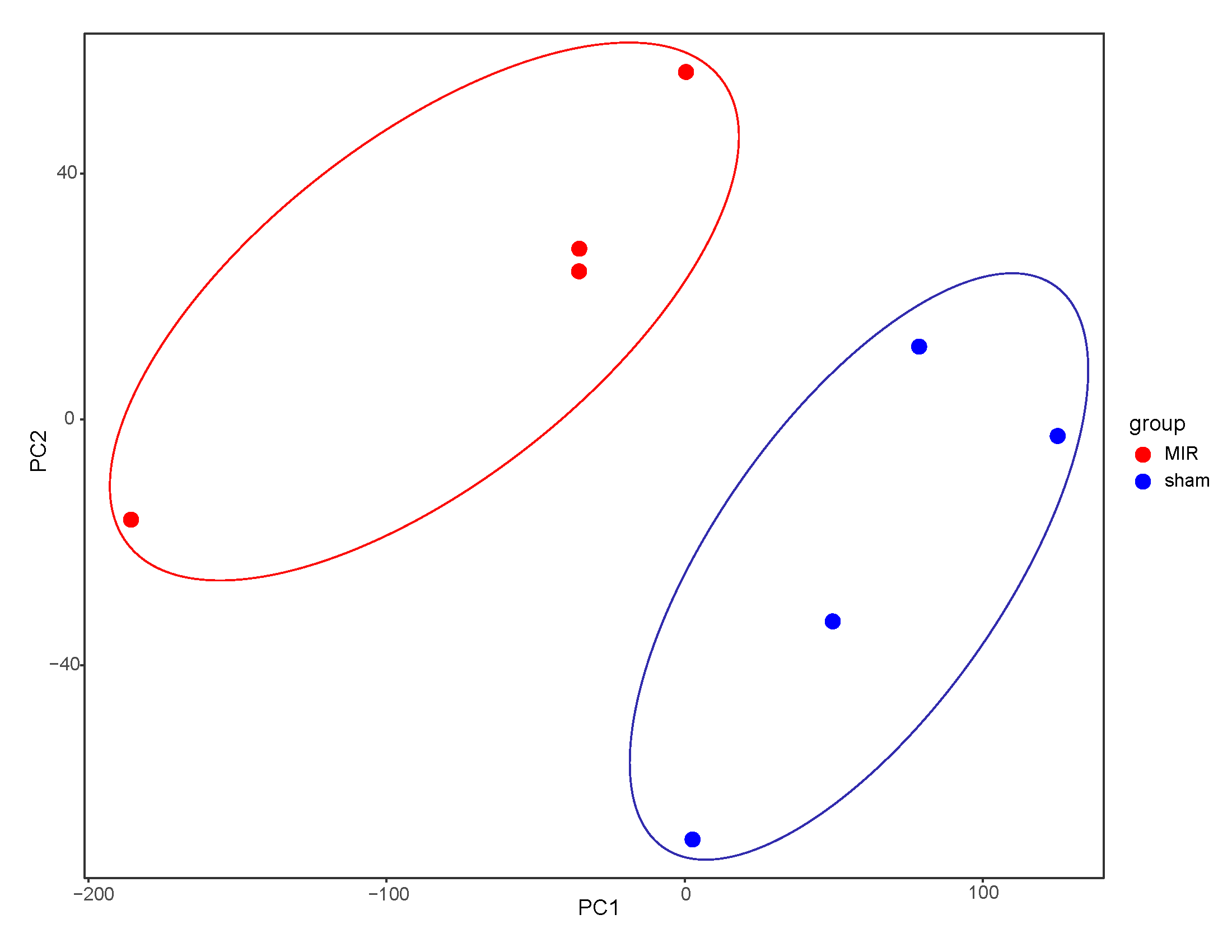


**Supplementary Figure 1.** PCA plot of GSE168610 RNA-seq data according to gene expression level.
