# Supplementary material for: Development and Validation of Robust Ferroptosis-Related Genes in Myocardial Ischemia-Reperfusion Injury
Source: J Cardiovasc Dev Dis. 2023 Aug 12;10(8):344. doi: 10.3390/jcdd10080344 (PMC10455596; doi:10.3390/jcdd10080344)
Supplement: Supplementary file 1 [file jcdd-10-00344-s001.zip › supplementary files/Additional file 2 (ST1).docx]

**Supplementary TABLE 1 |** GEO datasets used in this article.

| Datasets to identify DEFRGs | | |
| --- | --- | --- |
| GEO Series | Sample | Group |
| GSE4105 | GSM93898 | Sham |
|  | GSM93899 | Sham |
|  | GSM93900 | Sham |
|  | GSM93901 | Sham |
|  | GSM93902 | Sham |
|  | GSM93903 | Sham |
|  | GSM93881 | MI-R |
|  | GSM93893 | MI-R |
|  | GSM93894 | MI-R |
|  | GSM93895 | MI-R |
|  | GSM93896 | MI-R |
|  | GSM93897 | MI-R |
| GSE61592 | GSM1508996 | Sham |
|  | GSM1508997 | Sham |
|  | GSM1508998 | Sham |
|  | GSM1508999 | MI-R |
|  | GSM1509000 | MI-R |
|  | GSM1509001 | MI-R |
| GSE83472 | GSM2203979 | Sham |
|  | GSM2203980 | Sham |
|  | GSM2203983 | Sham |
|  | GSM2203984 | Sham |
|  | GSM2203977 | MI-R |
|  | GSM2203978 | MI-R |
|  | GSM2203982 | MI-R |
|  | GSM2203989 | MI-R |
| Datasets to validate hub gene | | |
| GEO Series | Sample | Group |
| GSE168610 | GSM5150637 | Sham |
|  | GSM5150638 | Sham |
|  | GSM5150639 | Sham |
|  | GSM5150640 | Sham |
|  | GSM5150629 | MI-R |
|  | GSM5150630 | MI-R |
|  | GSM5150631 | MI-R |
|  | GSM5150632 | MI-R |
