# Supplementary material for: Development and Validation of Robust Ferroptosis-Related Genes in Myocardial Ischemia-Reperfusion Injury
Source: J Cardiovasc Dev Dis. 2023 Aug 12;10(8):344. doi: 10.3390/jcdd10080344 (PMC10455596; doi:10.3390/jcdd10080344)
Supplement: Supplementary file 1 [file jcdd-10-00344-s001.zip › supplementary files/Additional file 3 (ST2).docx]

**Supplementary TABLE 2 |** Primers of ferroptosis DEGs used for qRT-PCR.

| **Gene name** | **Species** | **Primer Sequence** | |
| --- | --- | --- | --- |
| Atf3 | Rat | Forward Primer | CGCTGGTGTTTGAGGATTTT |
|  |  | Reverse Primer | CTGACTCCAGTGCAGACGAC |
| Hmox1 | Rat | Forward Primer | AGCATGTCCCAGGATTTGTC |
|  |  | Reverse Primer | ACTGGGTTCTGCTTGTTTCG |
| Egfr | Rat | Forward Primer | GGCCTCTTCTGCGATTTCG |
|  |  | Reverse Primer | GCAGCTTGACCCTTCTCGG |
| Cd44 | Rat | Forward Primer | TCCCACTATGACACATATTGC |
|  |  | Reverse Primer | ACACCTTCTCCTACTGTTGAC |
| Vegfa | Rat | Forward Primer | GGGAGCAGAAAGCCCATGAA |
|  |  | Reverse Primer | GCTGGCTTTGGTGAGGTTTG |
| Xbp1 | Rat | Forward Primer | GAAAGAAAGCCCGGATGAGC |
|  |  | Reverse Primer | ATTCATCCCCAAGCGTGTCC |
| Gpx4 | Rat | Forward Primer | AATCCTGGCCTTCCCTTGCA |
|  |  | Reverse Primer | GCCCTTGGGCTGGACTTTCA |
| Asns | Rat | Forward Primer | CCATCACTGTACGGATGAACCA |
|  |  | Reverse Primer | CGGTCAGTCATCAAGCGTTTC |
| Brd4 | Rat | Forward Primer | GGACGAGGGAGGAAAGAAACA |
|  |  | Reverse Primer | AGGAAAGGGGTGAGTTGTGG |
| Actb | Rat | Forward Primer | CGAGCGCGGCTACAGCTT |
|  |  | Reverse Primer | TCCTTAATGTCACGCACGATTT |
| Atf3 | Mouse | Forward Primer | GAGGATTTTGCTAACCTGACACC |
|  |  | Reverse Primer | TTGACGGTAACTGACTCCAGC |
| Hmox1 | Mouse | Forward Primer | GATAGAGCGCAACAAGCAGAA |
|  |  | Reverse Primer | CAGTGAGGCCCATACCAGAAG |
| Egfr | Mouse | Forward Primer | GCCATCTGGGCCAAAGATACC |
|  |  | Reverse Primer | GTCTTCGCATGAATAGGCCAAT |
| Cd44 | Mouse | Forward Primer | TCTGCCATCTAGCACTAAGAGC |
|  |  | Reverse Primer | GTCTGGGTATTGAAAGGTGTAGC |
| Vegfa | Mouse | Forward Primer | GCACATAGAGAGAATGAGCTTCC |
|  |  | Reverse Primer | CTCCGCTCTGAACAAGGCT |
| Xbp1 | Mouse | Forward Primer | AGCAGCAAGTGGTGGATTTG |
|  |  | Reverse Primer | GAGTTTTCTCCCGTAAAAGCTGA |
| Gpx4 | Mouse | Forward Primer | TCTGTGTAAATGGGGACGATGC |
|  |  | Reverse Primer | TCTCTATCACCTGGGGCTCCTC |
| Asns | Mouse | Forward Primer | GCAGTGTCTGAGTGCGATGAA |
|  |  | Reverse Primer | TCTTATCGGCTGCATTCCAAAC |
| Brd4 | Mouse | Forward Primer | AGTGTCTTTGACCCTATTAGCCA |
|  |  | Reverse Primer | CATGCTGGTTGAGATGGGG |
| Actb | Mouse | Forward Primer | TCAAGATCATTGCTCCTCCTGAG |
|  |  | Reverse Primer | ACATCTGCTGGAAGGTGGACA |
