# Supplementary material for: Development and Validation of Robust Ferroptosis-Related Genes in Myocardial Ischemia-Reperfusion Injury
Source: J Cardiovasc Dev Dis. 2023 Aug 12;10(8):344. doi: 10.3390/jcdd10080344 (PMC10455596; doi:10.3390/jcdd10080344)
Supplement: Supplementary file 1 [file jcdd-10-00344-s001.zip › supplementary files/Additional file 4 (ST3).docx]

**Supplementary TABLE 3 |** Classification of the DEFRGs.

| Marker (n=10) | Driver (n=7) | Suppressor (n=4) |
| --- | --- | --- |
| Capg, Asns, Vldlr, Psat1, Xbp1, Hmox1, Atf3, Vegfa, Gpx4, Ripk1 | Hmox1, Lpin1, Atf3, Cs, Scp2, Prkaa2, Egfr | Cd44, Hmox1, Gpx4, Brd4 |

Marker (n = 10) + Driver (n = 7) + Suppressor (n = 4) = 21, which was larger than the ferroptosis DEGs count (17), because of 3 multi-annotated genes (underlined) as shown in the Venn diagram.
