# Supplementary material for: Development and Validation of Robust Ferroptosis-Related Genes in Myocardial Ischemia-Reperfusion Injury
Source: J Cardiovasc Dev Dis. 2023 Aug 12;10(8):344. doi: 10.3390/jcdd10080344 (PMC10455596; doi:10.3390/jcdd10080344)
Supplement: Supplementary file 1 [file jcdd-10-00344-s001.zip › supplementary files/Additional file 5 (ST4).docx]

**Supplementary TABLE 4 |** DEFRGs including 10 upregulated and 7 downregulated genes in MI-R.

| Gene symbol | Description | GSE4105 | | GSE61592 | | GSE83472 | |
| --- | --- | --- | --- | --- | --- | --- | --- |
|  |  | log2FC | P.Value | log2FC | P.Value | log2FC | P.Value |
| Capg | Capping actin protein, gelsolin like | 0.820873 | 0.000197 | 2.912465 | 6.27E-14 | 0.852790 | 0.017121 |
| Asns | Asparagine synthetase (glutamine-hydrolyzing) | 0.489502 | 0.007989 | 1.656295 | 4.87E-10 | 0.822100 | 0.010341 |
| Vldlr | Very low density lipoprotein receptor | -0.279995 | 0.026484 | -2.324696 | 9.81E-14 | -0.740603 | 0.025060 |
| Psat1 | Phosphoserine aminotransferase 1 | 1.546980 | 0.005569 | 1.828973 | 5.72E-11 | 0.709969 | 0.010471 |
| Xbp1 | X-box binding protein 1 | 0.412075 | 0.008979 | 1.135744 | 9.23E-10 | 0.700977 | 0.012407 |
| Cd44 | CD44 molecule (Indian blood group) | 0.896190 | 0.000180 | 2.034241 | 9.08E-11 | 0.624994 | 0.025425 |
| Hmox1 | Heme oxygenase 1 | 1.550994 | 0.001440 | 3.302202 | 1.25E-09 | 0.476230 | 0.026799 |
| Lpin1 | Lipin 1 | -0.529400 | 0.004794 | -2.374641 | 2.02E-13 | -0.424813 | 0.017618 |
| Atf3 | Activating transcription factor 3 | 1.570235 | 0.013543 | 0.642502 | 6.26E-06 | 0.418002 | 0.030481 |
| Vegfa | Vascular endothelial growth factor A | -0.409595 | 0.004665 | -2.061900 | 3.68E-12 | -0.373457 | 0.000885 |
| Gpx4 | Glutathione peroxidase 4 | -0.215456 | 0.048128 | -1.078140 | 5.25E-10 | -0.332987 | 0.018277 |
| Cs | Citrate synthase | -0.303727 | 0.030672 | -1.739489 | 2.91E-11 | -0.321794 | 0.040874 |
| Scp2 | Sterol carrier protein 2 | -0.344961 | 0.017149 | -1.183173 | 2.72E-10 | -0.289011 | 0.027004 |
| Prkaa2 | Protein kinase AMP-activated catalytic subunit alpha 2 | -0.782153 | 0.011002 | -1.463266 | 9.39E-08 | -0.272087 | 0.000541 |
| Brd4 | Bromodomain containing 4 | 0.237265 | 0.035634 | 0.434914 | 1.48E-06 | 0.239188 | 0.008958 |
| Egfr | Epidermal growth factor receptor | 1.290614 | 0.042215 | 1.057413 | 1.61E-09 | 0.126322 | 0.047935 |
| Ripk1 | Receptor interacting serine/threonine kinase 1 | 2.153469 | 0.000282 | 1.056803 | 1.11E-08 | 0.119572 | 0.040766 |
