# Supplementary material for: Development and Validation of Robust Ferroptosis-Related Genes in Myocardial Ischemia-Reperfusion Injury
Source: J Cardiovasc Dev Dis. 2023 Aug 12;10(8):344. doi: 10.3390/jcdd10080344 (PMC10455596; doi:10.3390/jcdd10080344)
Supplement: Supplementary file 1 [file jcdd-10-00344-s001.zip › supplementary files/Additional file 6 (ST5).docx]

**Supplementary TABLE 5 |** Top 10 hub genes as ranked in CytoHubba.

| algorithms | MCC | | BottleNeck | | EPC | | EcCentricity | | Radiality | |
| --- | --- | --- | --- | --- | --- | --- | --- | --- | --- | --- |
| Rank | Name | Score | Name | Score | Name | Score | Name | Score | Name | Score |
| 1 | Egfr | 6 | Hmox1 | 11 | Hmox1 | 4.454 | Hmox1 | 0.333333 | Hmox1 | 5 |
| 2 | Hmox1 | 5 | Egfr | 10 | Egfr | 4.246 | Atf3 | 0.333333 | Egfr | 4.8 |
| 3 | Vegfa | 4 | Xbp1 | 3 | Vegfa | 3.857 | Xbp1 | 0.333333 | Vegfa | 4.6 |
| 4 | Atf3 | 4 | Asns | 2 | Xbp1 | 3.717 | Vegfa | 0.25 | Atf3 | 4.6 |
| 5 | Xbp1 | 4 | Cd44 | 1 | Atf3 | 3.673 | Asns | 0.25 | Xbp1 | 4.6 |
| 6 | Asns | 3 | Vegfa | 1 | Asns | 3.246 | Gpx4 | 0.25 | Gpx4 | 4.1 |
| 7 | Cd44 | 2 | Gpx4 | 1 | Cd44 | 3.165 | Egfr | 0.25 | Cd44 | 4 |
| 8 | Gpx4 | 1 | Psat1 | 1 | Gpx4 | 2.513 | Cd44 | 0.2 | Asns | 4 |
| 9 | Psat1 | 1 | Atf3 | 1 | Vldlr | 2.452 | Psat1 | 0.2 | Brd4 | 3.9 |
| 10 | Brd4 | 1 | Brd4 | 1 | Brd4 | 2.442 | Brd4 | 0.2 | Vldlr | 3.9 |
