# Supplementary material for: Development and Validation of Robust Ferroptosis-Related Genes in Myocardial Ischemia-Reperfusion Injury
Source: J Cardiovasc Dev Dis. 2023 Aug 12;10(8):344. doi: 10.3390/jcdd10080344 (PMC10455596; doi:10.3390/jcdd10080344)
Supplement: Supplementary file 1 [file jcdd-10-00344-s001.zip › supplementary files/Additional file 7 (ST6).docx]

**Supplementary TABLE 6 |** P.Values of hub gene expression in GSE168610.

| Gene symbol | Description | GSE168610 | |
| --- | --- | --- | --- |
|  |  | log2FC | P.Value* |
| Egfr | Epidermal growth factor receptor | 0.605649 | 0.001650 |
| Hmox1 | Heme oxygenase 1 | 3.982869 | 0.000000 |
| Vegfa | Vascular endothelial growth factor A | -0.172230 | 0.221378 |
| Atf3 | Activating transcription factor 3 | 1.981713 | 0.002028 |
| Xbp1 | X-box binding protein 1 | 0.386498 | 0.025293 |
| Asns | Asparagine synthetase (glutamine-hydrolyzing) | 0.711010 | 0.011175 |
| Cd44 | CD44 molecule (Indian blood group) | 1.820289 | 0.000000 |
| Gpx4 | Glutathione peroxidase 4 | -0.168243 | 0.138192 |
| Brd4 | Bromodomain containing 4 | 0.193628 | 0.083464 |

* Analyzed groups were compared using the Wilcoxon test.
