# Supplementary material for: Development and Validation of Robust Ferroptosis-Related Genes in Myocardial Ischemia-Reperfusion Injury
Source: J Cardiovasc Dev Dis. 2023 Aug 12;10(8):344. doi: 10.3390/jcdd10080344 (PMC10455596; doi:10.3390/jcdd10080344)
Supplement: Supplementary file 1 [file jcdd-10-00344-s001.zip › supplementary files/Additional file 8 (ST7).docx]

**Supplementary TABLE 7 |** Statistical information.

| Figure | Panel | Number | Shapiro-Wilk normality test | Statistical test |
| --- | --- | --- | --- | --- |
| Figure 6 | A | 4;4 | NA | Wilcoxon test |
| Figure 7 | A-Atf3 | 3;3 | passed | unpaired t-test |
|  | A-Hmox1 | 3;3 | passed | unpaired t-test |
|  | A-Egfr | 3;3 | passed | unpaired t-test |
|  | A-Cd44 | 3;3 | passed | unpaired t-test |
|  | A-Vegfa | 3;3 | passed | unpaired t-test |
|  | A-Xbp1 | 3;3 | passed | unpaired t-test |
|  | A-Gpx4 | 3;3 | NA | Mann-Whitney test |
|  | A-Asns | 3;3 | NA | Mann-Whitney test |
|  | A-Brd4 | 3;3 | passed | unpaired t-test |
|  | B-Hmox1 | 4;4 | passed | unpaired t-test |
|  | B-Atf3 | 4;4 | passed | unpaired t-test |
|  | D | 3;3 | passed | unpaired t-test |
| Figure 8 | A-EF | 4;4 | passed | unpaired t-test |
|  | A-FS | 4;4 | passed | unpaired t-test |
|  | C-Infract size | 3;3 | NA | Mann-Whitney test |
|  | C-Histopathological scoring | 3;3 | NA | Mann-Whitney test |
|  | E-GSH | 3;3 | passed | unpaired t-test |
|  | E-GSH/GSSG | 3;3 | passed | unpaired t-test |
| Figure 9 | A-Atf3 | 4;4 | passed | unpaired t-test |
|  | A-Hmox1 | 4;4 | passed | unpaired t-test |
|  | A-Egfr | 4;4 | passed | unpaired t-test |
|  | A-Cd44 | 4;4 | passed | unpaired t-test |
|  | A-Vegfa | 4;4 | passed | unpaired t-test |
|  | A-Xbp1 | 4;4 | passed | unpaired t-test |
|  | A-Gpx4 | 4;4 | passed | unpaired t-test |
|  | A-Asns | 4;4 | passed | unpaired t-test |
|  | A-Brd4 | 4;4 | passed | unpaired t-test |
|  | C-Hmox1 | 4;4 | passed | unpaired t-test |
|  | C-Atf3 | 4;4 | passed | unpaired t-test |
|  | D-Hmox1 | 5;5 | passed | unpaired t-test |
|  | D-Atf3 | 5;5 | passed | unpaired t-test |
| Figure 10 | B-CD45+ | 3;3 | passed | unpaired t-test |
|  | B-CD19+ | 3;3 | passed | unpaired t-test |
|  | B-M2 | 3;3 | passed | unpaired t-test |
|  | B-M1 | 3;3 | passed | unpaired t-test |
